# The Ordered Structures Formed by Janus-like Particles on a Triangular Lattice

**DOI:** 10.3390/molecules29215215

**Published:** 2024-11-04

**Authors:** Andrzej Patrykiejew

**Affiliations:** Department of Theoretical Chemistry, Institute of Chemical Sciences, Faculty of Chemistry, Maria Curie-Skłodowska University, 20-031 Lublin, Poland; andrzej.patrykiejew@mail.umcs.pl

**Keywords:** ordered structures, lattice model, orientation-dependent interactions, Monte Carlo simulation, phase transitions

## Abstract

The formation of ordered structures by Janus-like particles, composed of two parts (A and B), with orientation-dependent interactions on a triangular lattice was studied using Monte Carlo methods. The assumed lattice model allows each particle to take on one of the six orientations. The interaction between the A parts of neighboring particles was assumed to be attractive, while the AB and BB interactions were assumed to be repulsive. Moreover, it was assumed that the interaction between a pair of neighboring particles depended on the degrees to which their AA, AB, and BB parts face each other. It was shown that several ordered phases of different densities and structures may appear, depending on the magnitudes of AB and BB interactions. In particular, we found several structures composed of small clusters consisting of three (OT), four (OR), and seven (*S*) particles, surrounded by empty sites, the lamellar phases (OL, $OL_{1}$, and $OL_{3}$), the structures with hexagonal symmetry ($R_{3\times 3}$ and *K*), as well as the structures with more complex symmetry ($R_{5\times 5}$ and LAD). Several phase diagrams were evaluated, which demonstrated that the stability regions of different ordered phases are primarily determined by the strengths of repulsive AB and BB interactions.

## 1. Introduction

The surface of Janus particles is composed of two chemically different patches, A and B [1,2]. The surface chemical anisotropy, which can be tuned by appropriate functionalization, results in orientation-dependent interactions between the particles. Both the size and the chemical composition of patches influence the self-assembly and ordering of Janus particles in two- and three-dimensional systems [3,4,5,6,7,8]. At low and moderate densities, the formation of micelles, vesicles, and worm-like clusters have been observed [9,10,11]. It has also been shown that Janus particles may form crystals of different structure and density [11].

The behavior of dense two-dimensional systems of Janus particles has been studied by several authors [3,12,13,14]. Shin and Schweizer [3] used the Kern–Frenkel model [15], and developed a version of self-consistent phonon theory, which predicted the formation of different orientationally ordered hexagonal phases. The structure of these phases was found to be primarily determined by the so-called Janus balance [16], which is determined by the size of the attractive patch. Shin and Schweizer also showed that dense systems of Janus particles undergo phase transitions between different orientationally ordered phases. Similar structures were observed by Iwashita and Kimura [4]. On the other hand, experimental study and Monte Carlo simulation of Jiang et al. [5] demonstrated the formation of a glass-like phase, instead of a theoretically predicted zigzag phase [3].

In our recent paper [13], we studied orientational order–disorder transitions in close-packed two-dimensional systems of Janus particles, using the lattice model which allows each particle to take on one of the six orientations on a triangular lattice. It was demonstrated that the nature of the orientational order–disorder transition is entirely determined by the sign of the parameter $\epsilon =u_{AA}+u_{BB}-2u_{AB}$, where $u_{AA}$, $u_{AB}$, and $u_{BB}$ are the energies of interaction between the nearest neighbor pairs with the AA, AB, or BB halves facing each other. When ϵ<0, the systems order into the zigzag lamellar phase, and the order–disorder transition belongs to the universality class of the three-state Potts model [17]. On the other hand, when ϵ>0, the ordered phase structure is different. The lattice can be decomposed into four sublattices, and the orientations of particles on three of them are fixed, while the particles occupying the fourth sublattice are free to rotate. This phase was shown to undergo the order–disorder transition belonging to the universality class of the four-state Potts model [17].

The phase behavior of systems with non-repulsive AA, AB, and BB interactions has already been discussed in our earlier works [14,18]. It was shown that in the case of strongly anisotropic interactions, the gas–liquid transition may be suppressed and replaced by the first-order transition between the low-density fluid and the high-density ordered lamellar zigzag phase. This transition terminates at the tricritical point, marking the onset of a continuous transition between the fluid and lamellar phases. When the AB and/or BB attraction increases, it leads to a gradual decrease in the stability of the lamellar phase, and the dilute gas-like phase may condense into the ordered high-density lamellar phase only at sufficiently low temperatures, below the critical endpoint temperature, $T_{cep}$, marking the onset of continuous transition between the disordered lamellar fluid and the ordered lamellar phase. Upon the increase in temperature, the continuous liquid-ordered phase transition takes place at gradually increasing density and temperature. The transition terminates at the temperature of $T_{o}$, at which the order–disorder transition occurs in the close-packed system at a density of ρ=1.0.

At temperatures above the critical endpoint, the transition between a dilute gas and the disordered lamellar liquid takes place and terminates at the usual critical point.

In Ref. [14], we considered the particular system with $u_{AA}=-1.0$ and $u_{AB}=u_{BB}=0$, i.e., with the interaction potential similar to that proposed by Kern and Frenkel [15]. Two versions of the model were studied. In the first version, the strength of attractive AA interaction was assumed to depend on the degree to which the A parts of neighboring particles overlap. In the second version, the interaction energy between the A halves was assumed to be the same for any mutual orientations of adjacent particles, in which the A patches overlap to any extent. It was demonstrated that these two versions lead to qualitatively different results. In the first case, the self-assembly promotes the formation of different, partially ordered, stripped structures, depending on the density and temperature. In particular, it was shown that the condensation of a dilute gas leads directly to the high-density ordered zigzag phase at sufficiently low temperatures. At intermediate temperatures, the system undergoes two first-order phase transitions. The first transition is the condensation of the gas phase into the partially ordered phase ($Z_{2}$), consisting of kinked stripes predominantly oriented along two out of three lattice axes. The second transition occurs between the $Z_{2}$ phase and the high-density well-ordered lamellar phase. At still higher temperatures, only one continuous transition between the disordered fluid and the ordered lamellar phase was observed. In the second version of the model, only one discontinuous transition at low temperatures was found. This transition occurs between a dilute gas-like phase and the ordered phase of the density 6/7, and the structure corresponding to the Kagome lattice. A further increase in density triggers the reorientation of particles and leads to the formation of a glass-like structure, similar to that observed by Jiang et al. [5].

Unlike in our previous papers [13,14,18], where the interactions were assumed to be non-repulsive, here, we focused on systems with repulsive AB and/or BB interactions, in which the limited valence is expected to promote the formation of ordered phases characterized by different densities. It should be emphasized that such systems have not been studied so far. The main goal was to elucidate the role of orientation-dependent interactions in the formation of ordered phases characterized by different symmetries and densities, and to determine their stability. To this end, we used Monte Carlo simulation methods in the canonical and grand canonical ensembles to study the lattice gas model considered in Refs. [13,14,18]. Three series of systems were studied, assuming that the AA interaction is attractive and fixed. In the first series, the AB and BB interactions were assumed to be the same, $u_{AB}=u_{BB}=u^{*}$, and varied between 0.1 and 1.0. In the second (the third) series, $u_{AB}$ ($u_{BB}$) was assumed to be equal to zero, while $u_{BB}$ ($u_{AB}$) varied between 0.1 and 3.0.

The behavior of systems discussed here is of interest since it is plausible that the appropriate functionalization of surfaces may lead to the synthesis of particles with properties similar to those considered in this work.

## 2. The Ground State Properties

The repulsive AB and BB interactions reduce the valence of particles and lead to the formation of ordered phases characterized by different structures and densities [4,19]. The AA contacts are always preferred, while the AB and BB contacts are disfavored. This situation is somewhat similar to isotropic lattice gas models with the attractive interaction between the first nearest neighbors, and the repulsive interactions at larger distances [20,21]. The ground state behavior of such lattice gas models has been studied by several authors [20,22,23,24,25], who demonstrated the formation of different ordered structures.

In this section, we demonstrate that the orientation-dependent nearest neighbor interactions lead to the formation of a large number of ordered phases. Some of them have been found in the lattice models with isotropic interactions [20,21], and several other structures appear only when the interactions are orientation-dependent.

At T=0, the grand potential is given by the minimum of the Hamiltonian, defined in Equation (7) in Section 4. The free energy density of any ordered phase α, characterized by the density $\rho_{\alpha }$, is given by

(1) $$\omega_{\alpha }\left(\mu \right)=\rho_{\alpha }\left(u_{\alpha }-\mu \right)$$

where $u_{\alpha }$ is the potential energy per particle. At a given chemical potential, the stable phase is the one that minimizes the free energy density, and the coexistence point between the phases, α and β, is located at

(2) $$\mu_{tr}\left(\alpha ,\beta \right)=\frac{u_{\beta }\rho_{\beta }-u_{\alpha }\rho_{\alpha }}{\rho_{\beta }-\rho_{\alpha }}\;\;\;,$$

with $\rho_{\beta }>\rho_{\alpha }$.

**Figure 1:**
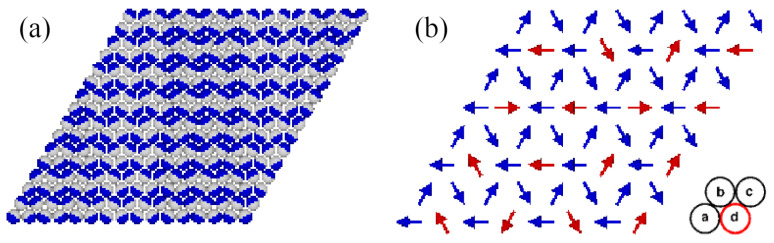
The structures of the close-packed phases LAM (**a**) and O_4_ (**b**). In part (**a**), parts A and B of each particle are shown in dark blue and light gray, respectively. In part (**b**), the orientations of particles located on different sublattices are marked by arrows pointing from part B to part A. The particles with fixed orientations (blue arrows) occupy the sublattices a, b, and c, and the freely rotating particles (red arrows) occupy the sublattice d (red circle).

At T=0, the gas phase corresponds to ρ=0 (empty lattice) and $\omega_{G}=0$. On the other hand, the structure of the closed packed phase, with the density of ρ=1, was demonstrated [13] to depend on the sign of the parameter $\epsilon =u_{AA}=u_{BB}-2u_{AB}$. When ϵ<0, the close-packed structure is the lamellar phase (LAM), which is composed of straight stripes consisting of rows in which the particles assume different orientations (Figure 1a). Within each pair of neighboring rows, the particles assume four different orientations, with the same probability of 0.25. The set of orientations in the LAM phase is determined by the orientation of occupied rows of sites.

When ϵ>0, the close-packed phase orders into the $O_{4}$ structure, schematically shown in Figure 1b. In this phase, the lattice can be decomposed into four interpenetrating sublattices. The orientations of particles located on the sublattices *a*, *b*, and *c* are fixed, while the particles on the fourth sublattice, d, are free to rotate. Again, the set of those fixed orientations depends on the orientation of the ordered phase relative to the lattice. Thus, the densities of particles with the fixed orientations are equal to 1/4+1/24, and the densities of particles with the remaining three orientations are equal to 1/24.

The LAM and $O_{4}$ phases have been demonstrated to undergo continuous order–disorder transitions belonging to different universality classes, and located at the temperature proportional to |ϵ| [13]. Thus, in the particular case of ϵ=0, the close-packed phase is orientationally disordered.

**Figure 2:**
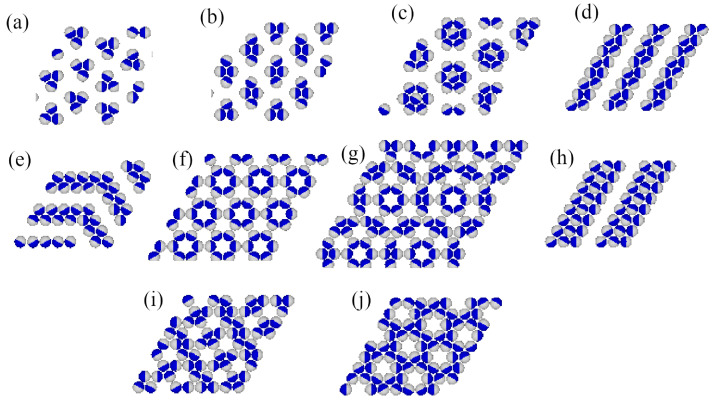
The parts of snapshots showing the observed ordered structures: ordered triangular (OT) (**a**), ordered romboidal (OR) (**b**), star-like (S) (**c**), ordered lamellar (OL) (**d**), $OL_{1}$ (**e**), $OL_{3}$ (**h**), $R_{3\times 3}$ (**f**), $R_{5\times 5}$ (**g**), ladder-like (LAD) (**i**), and kagome-like (*K*) (**j**). The A and B halves of the particles are shown in dark blue and light gray, respectively.

Grand canonical Monte Carlo simulations carried out at finite temperatures have shown that several ordered phases characterized by ρ<1 may appear. Figure 2 gives the fragments of snapshots demonstrating the structure of all detected ordered structures, and complete snapshots recorded during the simulations are given in the Supplementary Materials (Figure S1).

Knowing the structure of possible ordered phases, it was straightforward to determine their unit cells and to calculate the energies (per particle) at T=0 (see Table 1).

**Figure 3:**
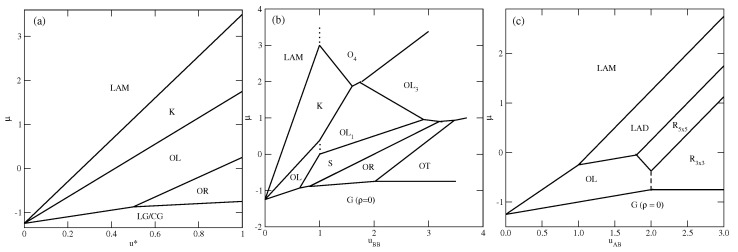
The ground state phase diagrams for the series with $u_{AB}=u_{BB}=u^{*}$ (**a**), with $u_{AB}=0$ (**b**), and $u_{BB}=0$ (**c**). The vertical dashed lines mark the crossover between the phases of the same density but of different symmetry.

In the first series, with $u_{AB}=u_{BB}=u^{*}$, only the ordered phases OR, OL, *K*, and LAM are stable at T=0. The OR phase is composed of rhomboidal clusters consisting of four particles each, surrounded by a layer of empty sites (Figure 2b). In a perfectly ordered OR phase, all rhombuses assume the same orientation relative to the lattice. For a given orientation of the OR phase, the particles located at the shorter diagonal are oriented such that their A parts face each other; hence, the probabilities of these two orientations are equal to 1/4. The probabilities of the remaining four orientations, associated with the particles located at the ends of the longer diagonal, are also the same, and equal to 1/8, since each particle can take on one of the two orientations with the same probability. The OL phase, of the density ρ=2/3, is composed of long parallel clusters, consisting of two rows of occupied sites, and separated by a single row of empty sites (Figure 2d). Within the stripes, the particles assume four different orientations with the probability of 1/4. The set of orientations appearing in the OL phase depends on the orientation of stripes relative to the lattice. The ordered phase *K*, of the density ρ=6/7, resembles the Kagome lattice (Figure 2j), but with empty sites separated by two rows of occupied sites. All six particles surrounding an empty site have their B halves directed towards the central empty site; hence, the probabilities of differently oriented particles are the same, and equal to 1/6. Finally, it should be noted that in the close-packed systems with $u_{AB}=u_{BB}$, the parameter ϵ is negative, and hence only the LAM phase appears. The ground state phase diagram for this series is given in Figure 3a. The OL and *K* phases are stable for any $u^{*}>0$, while the OR phase appears only for $u^{*}>0.5$. Here, we should mention that the lattice gas model with isotropic short-range attractive and long-range repulsive interactions was also found to exhibit the ordered OR and OL phases [20,21]. On the other hand, the *K* phase exists only due to the orientation-dependent interactions.

In the second series, with $u_{AB}=0$ and $u_{BB}>0$, the ground state behavior changes considerably (see Figure 3b), and several new ordered phases appear when the BB repulsion becomes stronger. Also, the close-packed phase assumes different structures when $u_{BB}$ is lower or higher than 1.0. The LAM phase is stable for $u_{BB}<1.0$, while the $O_{4}$ phase is stable when $u_{BB}>1$. The energy difference between these two phases is equal to 7ϵ/8, and is negative (positive) when $u_{BB}$ is lower (higher) than 1.0, since $u_{AA}=-1$ and $u_{AB}=0$. In the particular case of $u_{BB}=1$, the close-packed phase is orientationally disordered. It has also been found that the ordered phase with a density of ρ=2/3 assumes different structures when $u_{BB}$ is lower and higher than 1.0. For $u_{BB}<1.0$, it is the OL phase, shown in Figure 2d, while for $u_{BB}>1$, the phase $OL_{1}$ (Figure 2e) appears. These two structures are similar and consist of long clusters composed of two rows of occupied sites, separated by a single row of empty sites. However, in the $OL_{1}$ phase, the stripes are not straight and show several 120^o^ kinks, since the formation of kinks does not cost any energy. In addition, the particles within a single cluster take on only two orientations, and the particles in a single row assume the same orientation. The pair of orientations in each cluster may be different. Therefore, the $OL_{1}$ phase is highly degenerated. The difference between the energies of OL and $OL_{1}$ phases is the same as between the LAM and $O_{4}$ phases, and changes sign when ϵ=0. In this series, three other ordered phases, OT (Figure 2a), *S* (Figure 2c), and $OL_{3}$ (Figure 2h), appear in the ground state.

The ground state behavior of the third series, with $u_{BB}=0$ and $u_{AB}>0$, is different again, since the particles preferentially try to take on the orientations that minimize the number of AB contacts. This effect becomes stronger when $u_{AB}$ increases. The close-packed systems order into the LAM phase, since ϵ<0, but there are two different ordered phases with the density of ρ=2/3. The OL (cf. Figure 2d) phase is stable for $u_{AB}<2$, and the $R_{3\times 3}$ (cf. Figure 2f) phase is stable for $u_{AB}>2$. In the $R_{3\times 3}$ phase, the AB contacts do not appear at all; hence, its energy is independent of $u_{AB}$ and fixed $u_{R_{3\times 3}}=-3/4$, since $u_{AA}=-1.0$ and $u_{BB}=0$. This phase has two equivalent structures, shown in parts (f) and (g) of Figure S1 in the Supplementary Materials, which differ by the orientations of particles in the hexagons surrounding empty sites. Another ordered phase, $R_{5\times 5}$, with the density of ρ=18/25=0.72, has a rather complex structure consisting of hexagons, with the empty central site surrounded by rhomboidal clusters (see Figure 2g). We also found the ordered LAD phase (see Figure 2i), with a density of ρ=0.8, and a structure resembling a system of coupled ladders. This is well seen in the snapshot presented in Figure S1. The calculated ground state phase diagram for the series with $u_{BB}=0$ is shown in Figure 3c.

## 3. Finite Temperature Behavior

In this section, we present the results of Monte Carlo simulations carried out for the three series of systems. Our primary aim was to demonstrate that all of the ordered structures shown in Figure 2 appear at finite temperatures, and to estimate the phase diagrams for the selected systems.

### 3.1. The Series with $u_{AB}=u_{BB}=u^{*}$

From the ground state calculations, it follows that for $u^{*}\in \left[0.1,0.5\right]$, only the OL and *K* ordered structures should appear prior to the formation of the close-packed (ρ=1.0) LAM phase. When $u^{*}$ is greater than 0.5, the OR phase, with a density of ρ=0.5, is also expected to be present.

**Figure 4:**
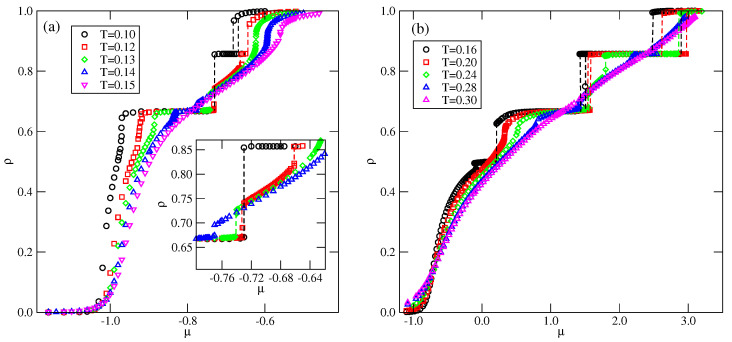
The examples of isotherms recorded for the systems with $u^{*}=0.1$ (**a**) and 0.8 (**b**), at different temperatures (given in the figure). The inset to part (**a**) shows the regions over which the OL−K and OL−LF transitions take place.

Figure 4 shows the examples of isotherms, recorded for $u^{*}$ lower and higher than 0.5. In the case of $u^{*}<0.5$ (see Figure 4a), the isotherms exhibit plateaus at ρ=2/3 and 6/7, which correspond to the OL and *K* ordered phases. The inset to Figure 4a shows that the first-order transition between the OL and *K* phases takes place only at sufficiently low temperatures, and splits into two transitions at higher temperatures. The first transition occurs between the OL ordered phase and the disordered lamellar fluid (LF). At a higher chemical potential, the transition between the disordered lamellar fluid and the ordered *K* phases takes place.

In the case of $u^{*}>0.5$ (Figure 4b), the isotherms recorded at sufficiently low temperatures show a plateau at ρ≈0.5, which corresponds to the OR phase. These results are quite consistent with the ground state predictions.

**Figure 5:**
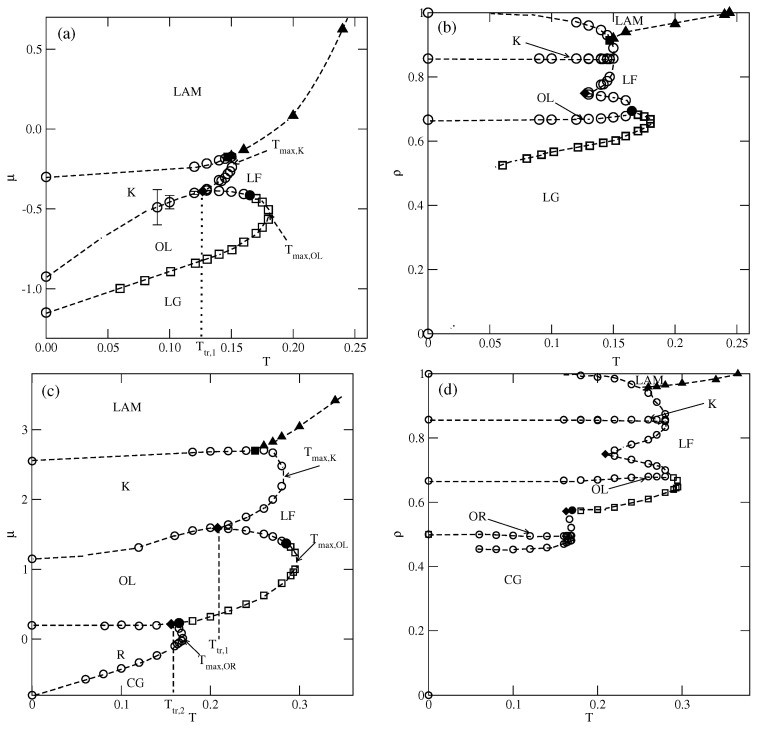
The estimated phase diagrams for $u^{*}=0.2$ (parts (**a**,**b**)) and 0.8 (parts (**c**,**d**)). Parts (**a**–**d**) show the T−μ (T−ρ) projections. Circles and squares mark the first-order and the second-order transitions, respectively. Only the second-order transition between the lamellar fluid (LF) and the LAM phase is marked by filled triangles.

We performed simulations for several values of $u^{*}$ between 0.1 and 1, and the estimated phase diagrams for $u^{*}=0.2$ and 0.8 are shown in Figure 5.

In the case of $u^{*}=0.2$, the first transition between the dilute lamellar gas (LG) (see Figure S2a in the Supplementary Materials) and the ordered OL phase was found to be continuous, even at the lowest temperature studied, equal to T=0.06 (cf. Figure 5a,b). The simulations at still lower temperatures suffered from very long-living metastable frozen states, and we could not obtain reliable results and resolve the question of whether the continuous LG−OL transition terminates at the tricritical point at a finite temperature, or the first-order LG−OL transition occurs only at T=0. It should be emphasized that similar results have been obtained for other systems with $u^{*}\leq 0.5$. In the case of $u^{*}=0.2$, the OL phase is stable at the temperatures up to $T_{max,OL}$, equal to about 0.18. In order to confirm that $T_{max,OL}$ is the stability limit of the OL phase, we performed canonical ensemble simulation, in which the we used a perfectly ordered OL phase with a density of 2/3 obtained from the ground canonical simulation and the simulation cell of the size 72×72. The results confirmed the OL phase disorders via a continuous transition at the temperature of about 0.18.

On the other hand, the transition between the OL and *K* phases is discontinuous up to the triple point temperature, $T_{tr,1}$. Above the triple point, the OL phase undergoes a transition to the disordered lamellar fluid (LF) (see Figure S2b). This transition is discontinuous at the temperatures below the tricritical point temperature, $T_{trc,OL}$, marking the onset of the continuous OL−LF transition, which meets the continuous LG−OL transition at $T_{max,OL}$. Unlike in the case of the low-density LG phase, the high-density LF disordered phase consists of percolating clusters, and forms a sort of a random porous network (see Figure S2b).

The transition between the *K* and LAM ordered phases is discontinuous and terminates at the critical endpoint, $T_{cep}$. Above $T_{cep}$, the *K* phase undergoes a first-order transition to the disordered LF, and terminates at the temperature $T_{max,K}$, marking the stability limit of the *K* ordered phase. The critical endpoint is the onset of a continuous transition between the LF and the ordered LAM phases. Upon the increase in temperature, the transition occurs at gradually increasing density, and terminates at the temperature $T_{o}$, where the close-packed system undergoes the continuous orientational order–disorder transition. It was demonstrated [13] that $T_{o}$ linearly changes with ϵ, and for $u^{*}=0.2$, it is equal to about 0.245.

In the systems with $u^{*}>0.5$, the phase behavior changes, due to the formation of the OR phase (see Figure 5c,d). The dilute gas-like phase already shows a different structure. Instead of isolated lamellar clusters (cf. Figure S2a), it consists of isolated rhomboidal clusters (see Figure S3a). Upon the increase in density, the number of these clusters increases, leading to the formation of quite dense cluster fluid, CF (see Figure S3b–d). Ultimately, the cluster fluid undergoes a discontinuous transition to the well-ordered OR phase. This transition terminates at the stability limit of the OR structure, at $T_{max,OR}$. The canonical ensemble simulations with a starting configuration corresponding to a perfectly ordered OR phase show that the disordering of the OR phase occurs via a discontinuous transition at $T_{max,OR}$. Here, we should mention the results presented in the paper by N. Almarza et al. [21], in which the OR phase was found in the lattice model with the isotropic short-range attractive and long-range repulsive interactions. These authors showed that the disordering of the OR phase occurs via a discontinuous transition. At higher densities, the phase behavior of the systems with $u^{*}$ lower and higher than 0.5 is qualitatively the same.

**Figure 6:**
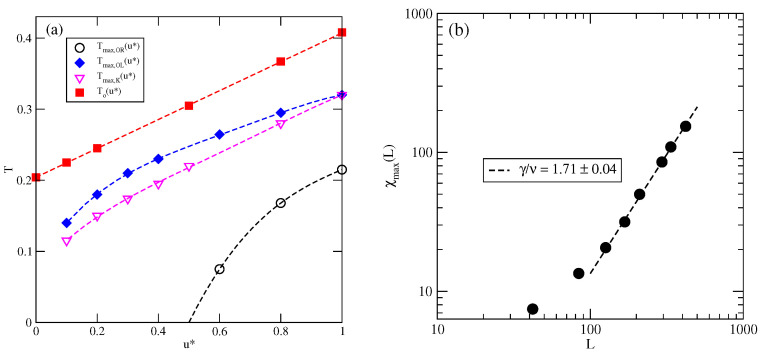
The changes in different characteristic temperatures (see the legend in the figure) with $u^{*}$ (**a**). The log–log plot of $\chi_{max}\left(L\right)$ versus *L* for the system with $u^{*}=0.1$ at T=0.14 (**b**).

The estimated phase diagrams for several values of $u^{*}$ allowed us to conclude that the stability of the ordered OR, OL, *K*, and LAM phases increases with $u^{*}$. Figure 6a shows the changes in $T_{max,OR}$, $T_{max,OL}$, and $T_{max,K}$ with $u^{*}$. Figure 6a also includes the temperature of the order–disorder transition in the close-packed LAM phase, $T_{o}\left(u^{*}\right)$. This transition belongs to the universality class of the three-state Potts model [13] and it can be expected that above the critical endpoint, the continuous LF−LAM transition also belongs to the same universality class. We attempted to confirm this prediction by calculating the density susceptibility for the system with $u^{*}=0.1$ at T=0.14, using simulation cells of different sizes. The finite size scaling theory [26] predicts that the density susceptibility maxima, $\chi_{max}\left(L\right)$, obey the power law

(3) $$\chi_{max}\left(L\right)\propto L^{\gamma /\nu }$$

with γ/ν=26/15≈1.733 [17]. We found that $\chi_{max}\left(L\right)$ does obey the above scaling relation, with γ/ν equal to 1.71±0.04 (Figure 6b). This result agrees quite well with the exact value.

### 3.2. The Series with $u_{AB}=0$

The ground state calculations for systems with $u_{AB}=0$ showed (cf. Figure 3b) the presence of OT (Figure 2a), *S* (Figure 2c), $OL_{1}$ (Figure 2e), and $OL_{3}$ (Figure 2h) ordered structures, apart of the OR, OL, and *K* phases. In addition, the structure of the closed packed phase changes from LAM to $O_{4}$, when $u_{BB}$ becomes greater than 1.0.

**Figure 7:**
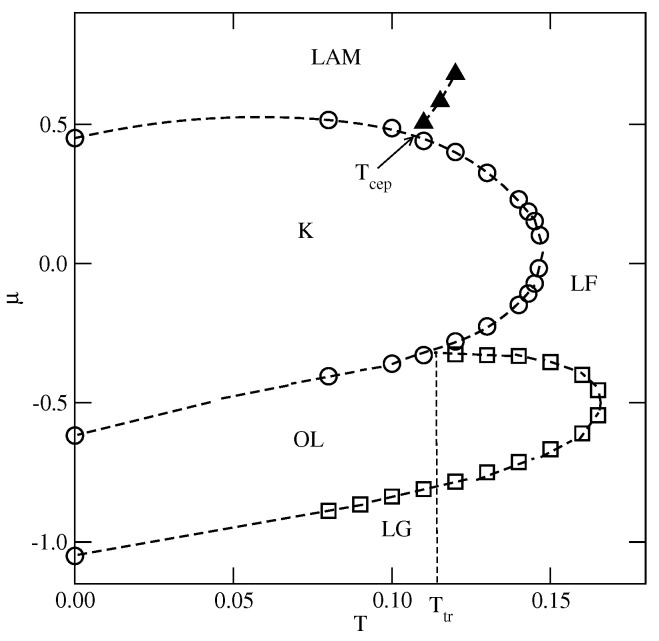
The estimated T−μ projection of the phase diagram for the system with $u_{AB}=0$ and $u_{BB}=0.4$. The first-order transitions are marked by circles, and the continuous order–disorder transition of the OL (LAM) phase is marked by squares (filled triangles).

Whenever $u_{BB}<0.65$, the phase behavior is qualitatively the same as in the already discussed series with $u^{*}<0.5$. Indeed, the T−μ projection of the phase diagram for the system with $u_{BB}=0.4$ (see Figure 7) is quite similar to that obtained for $u^{*}=0.2$ (cf. Figure 5a). In particular, the continuous LG−OL and OL−LF transitions terminate at $T_{max,OL}\approx 0.165$, the stability limit of the OL structure. The OL−K transition is discontinuous, and terminates at the triple point, $T_{tr,1}$. Also, the *K* phase undergoes a discontinuous disordering transition into the LF.

**Figure 8:**
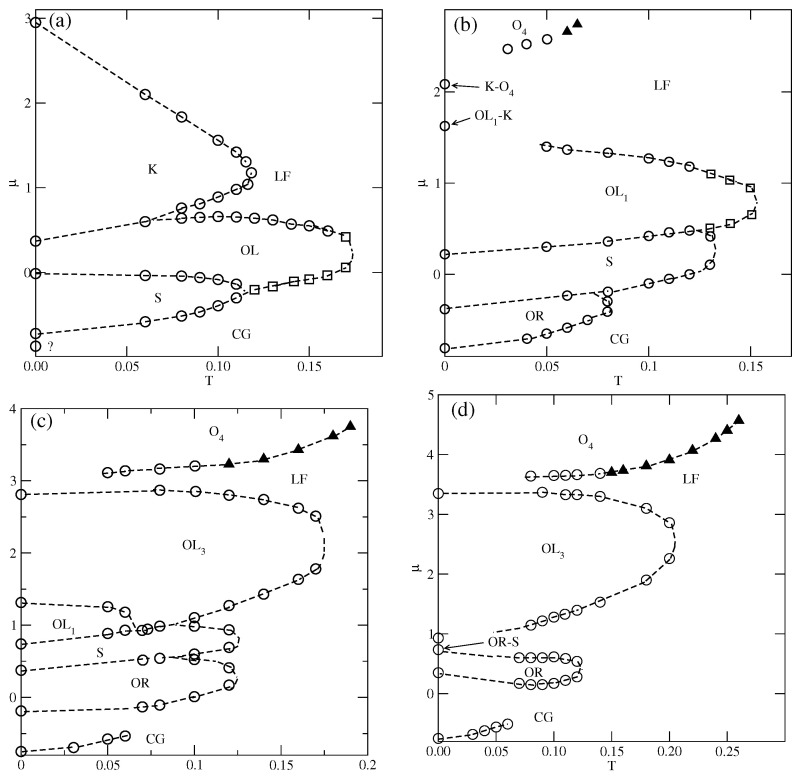
The T−μ projections of the phase diagrams for the systems with $u_{AB}=0$ and different values of $u_{BB}$ equal to 1.0 (**a**), 1.5 (**b**), 2.5 (**c**), and 3.0 (**d**). The boundaries of first-order (second-order) transitions are marked by circles (squares). The continuous transition between the disordered lamellar fluid and the $O_{4}$ phase is marked by filled triangles.

The situation changes when the repulsive BB interaction becomes stronger, when $u_{BB}>0.65$, since new ordered phases are expected to appear and be stable at finite temperatures. The estimated phase diagrams for $u_{BB}$ equal to 1.0, 1.5, 2.5, and 3.0, are presented in Figure 8.

In the case of $u_{BB}=1.0$, the ordered OR phase is predicted to occur at T=0, but we did not observe it even at very low temperatures, down to T=0.04. Figure 8a shows the presence of *S*, OL, and *K* ordered phases. In the grand canonical simulations, the formation of the OR phase is likely to be hampered, since at T=0, the difference between the energies (per particle) of *S* and OR phases is small, and equal to about 0.018. We recall that in the series with $u_{AB}=u_{BB}$, the dilute disordered fluid consists of rhomboidal clusters (cf. Figure S3), and promotes the development of the OR phase. In the system with $u_{AB}=0$ and $u_{BB}=1.0$, the structure of a dilute disordered phase is different and changes with the density. The examples of configurations, recorded at T=0.04, in the region of densities in which the OR phase could be expected to appear, are shown in Figure S4. The snapshots demonstrate that the contribution of seven-particle clusters, characteristic of the *S* phase increases with density, while the contribution of four-particle rhomboidal clusters decreases. Consequently, this disordered phase undergoes the first-order transition directly to the ordered *S* phase. Here, we should note that the canonical ensemble simulation, with an extremely low temperature of 0.001, and the starting configuration being a perfectly ordered OR phase, has shown that this phase disorders at T≈0.067. On the other hand, the freezing run, starting at T=0.1, never leads to the recovery of the OR phase at low temperatures, but the same disordered structure is obtained from the grand canonical simulation.

**Figure 9:**
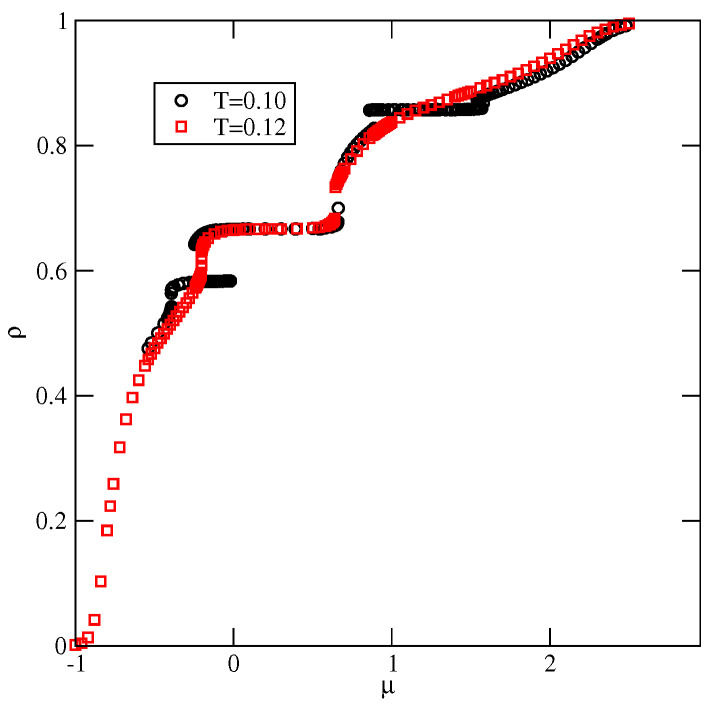
The isotherms for the system with $u_{BB}=1.0$, recorded at T=0.10 (circles) and 0.12 (squares).

Figure 9 shows the adsorption–desorption isotherms recorded at T=0.10 and 0.12, which demonstrate that at T=0.10, the disordered fluid condenses into the *S* phase, which undergoes the first-order transition to the OL phase. The isotherm at T=0.10 also exhibits a rather wide plateau, indicating the presence of the ordered *K* phase. On the other hand, at T=0.12, the *S* phase does not appear at all; the transition between the disordered fluid and the OL phase is continuous, and the OL phase undergoes another continuous transition to the high-density lamellar fluid, and the *K* phase is not present. Figure 9 also shows that in the high-density region, the isotherms do not exhibit any anomalies. Similarly, the densities of differently oriented particles, along the high-density parts of the isotherms at different temperatures, have shown that all orientations are equally probable. Thus, neither the LAM nor the $O_{4}$ ordered phase is formed.

The phase behavior changes when $u_{BB}=1.5$ (cf. Figure 8b). The OR phase shows considerably higher stability and is present at temperatures up to about 0.08. In this system, the energy difference between the *S* and OR phases is higher and equal to 0.0625, causing the low-density disordered phase to consist of mostly rhomboidal clusters, which allows for the formation of the OR phase.

**Figure 10:**
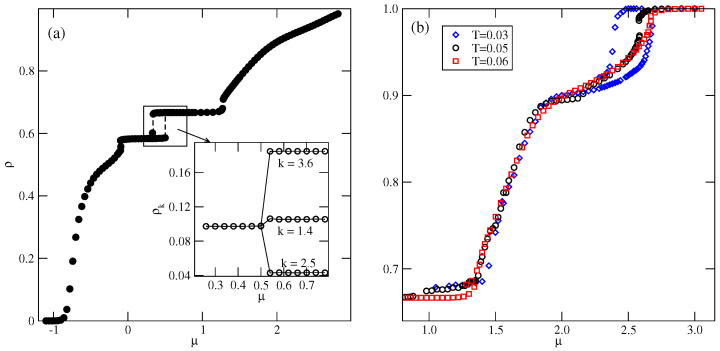
The isotherm at T=0.10, for the system with $u_{BB}=1.5$. The inset shows the densities of differently oriented particles in the vicinity of $S-OL_{1}$ transition along the adsorption branch of the isotherm (**a**). Part (**b**) shows the high-density parts of isotherms at different temperatures for the same system.

Also, the *S* and $OL_{1}$ phases are stable over a rather wide range of temperatures up to about 0.134 and 0.153, respectively. In the *S* phase, all orientations of particles are equally probable, and its presence was manifested by plateaus on isotherms. On the other hand, the $OL_{1}$ phase, of the same density as the OL phase, is characterized by different probabilities of particle orientations in the rows and assumes mutual orientations in which the A halves face each other in neighboring rows. Therefore, the pairs of orientations (1, 4), (2, 5), or (3, 6) should appear, but the probability of each pair may be different. Figure 10a presents the adsorption–desorption isotherm at T = 0.10, and the inset shows the changes in densities of differently oriented particles in the vicinity of the $S-OL_{1}$ transition, and confirm the predicted behavior of $\rho_{k}$ in the $OL_{1}$ phase.

**Figure 11:**
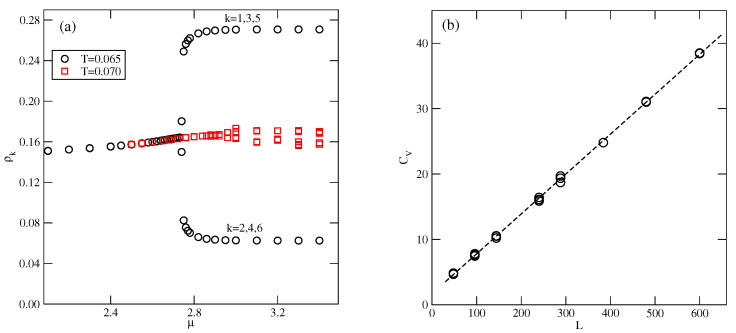
The changes in $\rho_{k}$ along the isotherms at T=0.065 and 0.07 (**a**), and the changes in the heat capacity maxima of the close-packed system for different simulation cell sizes (**b**), for $u_{BB}=1.5$.

Figure 10b presents the high-density parts of isotherms at T=0.03, 0.05, and 0.6, which do not show any trace of the *K* phase. Evidently, this ordered structure is stable only at extremely low temperatures. Also, the snapshots recorded along the isotherms did not show the presence of the ordered *K* phase. On the other hand, the isotherms in Figure 10b show the presence of a transition between the disordered dense phase and the ordered $O_{4}$ phase. This transition appears to be discontinuous at sufficiently low temperatures, up to about 0.05 and continuous at higher temperatures. This was confirmed by the behavior of heat capacity curves recorded along the T=0.05 and 0.06 isotherms. The simulations showed that the continuous $LF-O_{4}$ transition occurs at T=0.065, and is likely to terminate slightly below T=0.07. Figure 11a presents the changes in $\rho_{k}$ along the isotherms at T=0.065 and 0.07, and demonstrates that the $O_{4}$ phase does occur at T=0.065, but not at T=0.07. Our earlier study of closed packed systems [13] showed that in the case of $u_{BB}>1.0$, the systems order into the $O_{4}$ phase, and undergo the continuous disordering transition belonging to the universality class of the four-state Potts model. In the case of $u_{BB}=1.5$, this transition occurs at $T_{o}\approx 0.07$. Thus, the grand canonical ensemble calculations agree very well with this prediction. We also calculated the heat capacity curves for the system with ρ=1 using different sizes of the simulation cell. In the case of the four-state Potts model, the critical exponents ν and α are both equal to 2/3; hence, the heat capacity maxima, which obey the scaling law

(4) $$C_{max}\propto L^{\alpha /\nu }\;\;,$$

should linearly change with *L*. Indeed, the plot of $C_{max}$ versus *L*, given in Figure 11b, agrees with this prediction very well.

The phase diagram for the system with $u_{BB}=2.5$ (Figure 8c) shows the presence of all ordered phases predicted by the ground state considerations (cf. Figure 3b). The transition between the cluster gas and the ordered phase of the lowest density (OT) occurs at very low temperatures, and seems to terminate at the critical point. The disordered gas and the ordered phase consist of the same three-particle clusters; hence, there is a similarity between this transition and the gas–liquid transition in systems with isotropic interactions. The transitions between the ordered phases of higher densities demonstrate the re-entrant behavior, as in the systems with lower values of $u_{BB}$. Also, the transition leading to the $O_{4}$ phase changes order at the tricritical point.

In the case of $u_{BB}=3.0$ (see Figure 8d), the $OL_{1}$ phase does not appear, and the stability of the *S* phase is limited to very low temperatures, at which we were not able to obtain reliable results. In the systems with $u_{BB}=2.5$ and 3.0, we could not determine whether the $OL_{3}-O_{4}$ transition occurs at finite temperatures and terminates at the triple point, or occurs only at T=0.

### 3.3. $u_{BB}=0$

**Figure 12:**
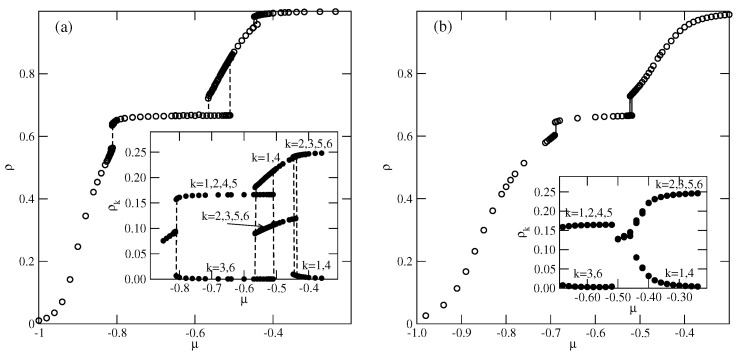
The isotherms for the system with $u_{BB}=0$ and $u_{AB}=0.5$, recorded at T=0.14 (**a**) and 0.17 (**b**). The insets show the changes in probabilities of differently oriented particles along the isotherms.

In the series with $u_{BB}=0$ and $u_{AB}\leq 1.0$, only the ordered phases OL and LAM are expected to form (cf. Figure 3c). Indeed, the low-temperature simulations demonstrated the formation of these two ordered phases. Parts (a) and (b) of Figure 12 present the examples of isotherms for the system with $u_{AB}=0.5$ at two different temperatures, and the insets show the changes in densities of differently oriented particles along the isotherms. At the temperature of 0.14 (Figure 12a), three discontinuous transitions are present. The first transition occurs between the disordered low-density lamellar fluid and the ordered OL phase. The second transition is between the OL and partially ordered phase, in which the meandering worm-like clusters are preferentially oriented along two lattice axes (see Figure S5). The inset to Figure 12a shows that the probabilities of the orientations with k=1 and 4 are high, while the probabilities of the remaining four orientations are considerably lower. The same partially ordered structure was observed at temperatures between 0.10 and 0.16. The third, also discontinuous, transition leads to the formation of the high-density LAM phase.

**Figure 13:**
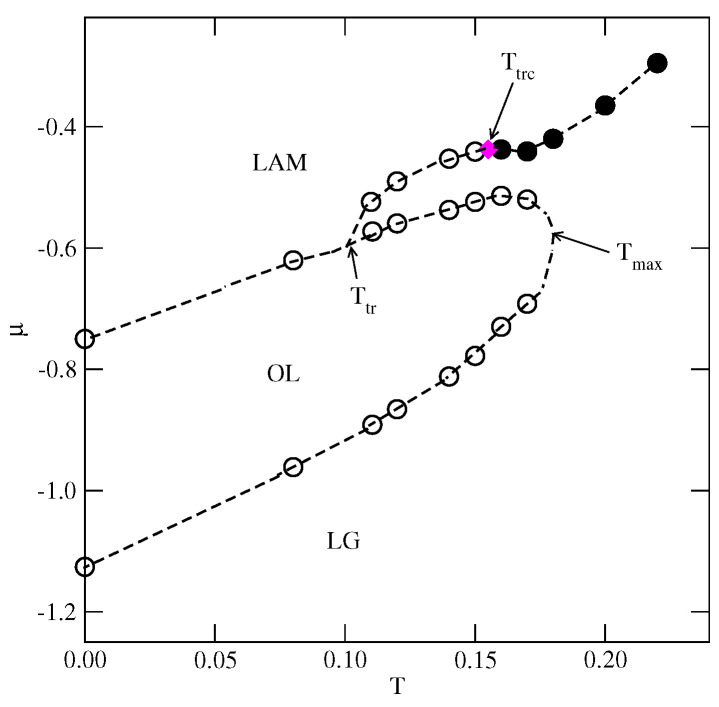
The estimated phase diagram for the system with $u_{BB}=0$ and $u_{AB}=0.5$. The first-order transitions are represented by empty circles, and the continuous transition between the disordered fluid and the LAM phases is marked by filled circles.The tricritical point is marked by the magenta diamond.

At T=0.17 (Figure 12b), only two discontinuous transitions are present. Again, the first transition leads to the formation of the OL phase, but the second transition leads to the disordered lamellar fluid, consisting of rather short and randomly oriented meandering worm-like clusters, and the same probabilities for all orientations (see Figure 12b). The isotherm at T=0.17 does not show any trace of transition between the lamellar fluid and the LAM phase. However, the changes in the densities of differently oriented particles (see the inset to Figure 12b) indicate the presence of a continuous transition between the disordered and LAM phases. From the simulations carried out at different temperatures, we estimated the phase diagram for this system, which is shown in Figure 13.

**Figure 14:**
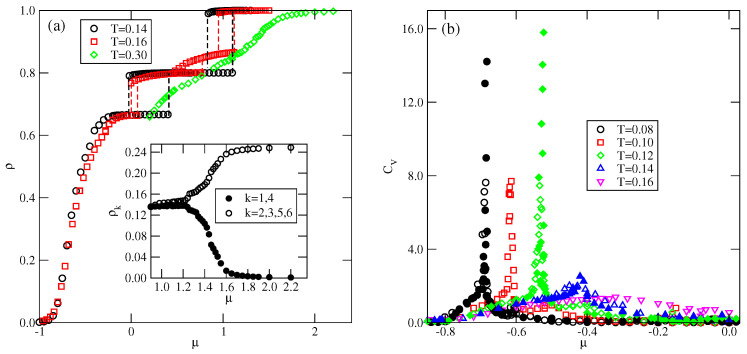
The isotherms (**a**) and the heat capacity (**b**) for the system with $u_{BB}=0$ and $u_{AB}=1.5$ recorded at different temperatures. The inset to part (**a**) shows the densities of differently oriented particles along the high-density part of the isotherm at T=0.30. Open and filled symbols in part (**b**) correspond to the results obtained using simulation cells with L=60 and L=90, respectively.

For $u_{AB}$ greater than 1.0 and lower than 1.8, the ground state predicts the appearance of the LAD phase, with a density of 0.8. Indeed, the simulations demonstrated the presence of this phase over a rather wide range of temperatures. Figure 14a presents the examples of ascending and descending isotherms for $u_{AB}=1.5$, which show the presence of an LAD phase, between the ordered OL and LAM phases.

These isotherms suggest that the transition between the low-density disordered lamellar fluid and the ordered OL phase is continuous. The calculated heat capacity curves at different temperatures, shown in Figure 14b, also demonstrate the appearance of peaks, with the size-dependent height. The results shown in Figure 14b support the expectation that the transition is continuous and indicate that it terminates at a certain temperature above 0.12, and below 0.14. It should be noted that at T=0, the transition between the gas and the OL phases is discontinuous. Therefore, the question arises as to whether the first-order transition occurs only in the ground state or also at finite temperatures. We were not able to resolve it, since the simulations at very low temperatures suffer from the long-living metastable frozen states.

The isotherms in Figure 14a also show that the OL−LAD and the LAD−LAM transitions are discontinuous at low temperatures, and accompanied by quite broad hysteresis loops due to long-living metastable states. Therefore, a direct estimation of transition points from the isotherms is subjected to large errors. A possible solution would be to calculate the free energies of the coexisting phases using the method of thermodynamic integration [27,28]. Since our study was qualitative in nature, we did not attempt such calculations. The isotherm at T=0.16 shows that the LAD−LAM transition splits into two transitions, due to the presence of an intervening dense disordered phase. At higher temperatures, the isotherms do not show any anomalies and the density gradually increases with the chemical potential. However, one expects the development of the LAM phase at sufficiently high densities. The inset to Figure 14a shows that at T=0.30, the dense disordered phase undergoes a continuous transition accompanied by the changes in densities of differently oriented particles characteristic of the LAM phase.

When $u_{AB}\geq 2.0$, the OL phase is replaced by the $R_{3\times 3}$ phase of the same density, but of a completely different structure (cf. Figure 2d,f). In addition, when $u_{AB}>1.8$, the ordered $R_{5\times 5}$ phase (cf. Figure 2g), with a density of ρ=0.72, is expected to appear before the formation of LAD phase.

**Figure 15:**
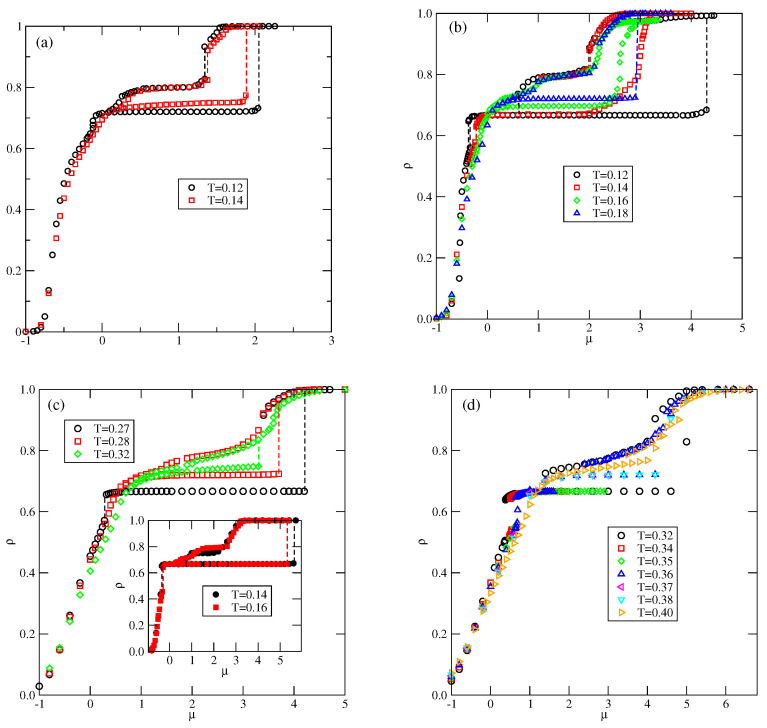
The isotherms for the systems with $u_{AB}=2.0$ (**a**), 2.5 (**b**), 3.0 (**c**), and 3.5 (**d**), recorded at different temperatures (given in the figure).

In the particular case of $u_{AB}=2.0$, neither the OL nor $R_{3\times 3}$ phases are expected to occur, since this value of $u_{AB}$ delimits the regions of their stability at T=0 (cf. Figure 3c). Indeed, the isotherms given in Figure 15a show that at the temperatures up to about 0.13, only the $R_{5\times 5}$ phase appears. A partially ordered $R_{5\times 5}$ phase is also present at *T* = 0.14. The $R_{5\times 5}$ phase undergoes a discontinuous transition directly to the LAM phase, without any trace of the LAD phase. The LAD phase appears only on descending isotherms and is not perfectly ordered. The recorded snapshots show the presence of large but differently oriented patches of the LAD phase.

Below, we present the results for the systems with $u_{AB}=2.5$, 3.0, and 3.5 of qualitatively the same ground state properties, but of different behavior at finite temperatures. Figure 15b,d present several adsorption–desorption isotherms for these three systems, and demonstrate that the increase in $u_{AB}$ primarily affects the stabilities of the $R_{3\times 3}$ and $R_{5\times 5}$ phases.

In the system with $u_{AB}=2.5$ (Figure 15b), the isotherms at temperatures lower than about 0.13 show the presence of a first-order transition between the low-density disordered phase and the well-ordered $R_{3\times 3}$ phase, followed by another first-order transition leading directly to the LAM phase. At the temperature equal to 0.14, the $R_{3\times 3}$ phase is still present, but stable over a considerably smaller range of the chemical potential, up to about 2.0. A further increase in μ causes the film density to increase continuously to high values, close to unity. At the temperatures between about 0.15 and 0.17, the isotherms reach a temperature-dependent plateau at ρ with densities between those of perfectly ordered $R_{3\times 3}$ and $R_{5\times 5}$ phases. The film structure consists of domains which exhibit elements of ordering characteristic of these two phases. In this temperature range, the density increases continuously and reaches high values. A further increase in temperature to T=0.18 causes only the well-ordered $R_{5\times 5}$ phase to be stable over a rather wide range of the chemical potential and undergoes a discontinuous transition to the LAM phase. The same behavior occurs at the temperatures of up to about 0.23. At higher temperatures, the $R_{5\times 5}$ phase loses its stability. Again, the descending parts of low-temperature isotherms show the defected LAD phase, which has never appeared on the ascending parts of isotherms. The lack of LAD ordering on ascending isotherms can be understood by taking into account the structure of the low-density disordered phase, which contains many hexagonal clusters, being the building blocks for the $R_{3\times 3}$ phase. On the other hand, when the initial structure corresponds to the LAM phase, a gradual decrease in density promotes the formation of LAD phase, but not the $R_{5\times 5}$ phase.

**Figure 16:**
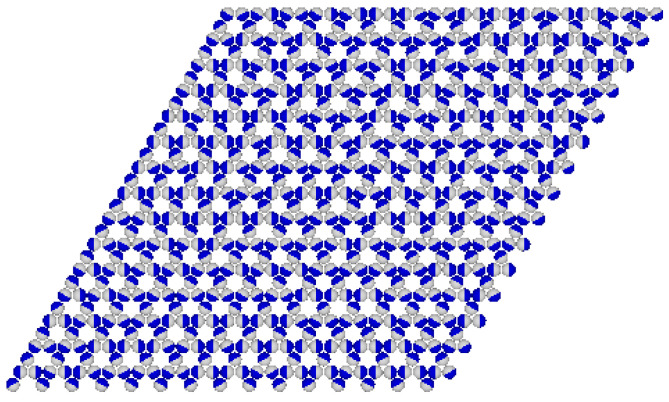
The snapshot for the system with $u_{AB}=3.0$ recorded at T=0.14 and μ=2.0.

A further increase in $u_{AB}$ to 3.0 leads to a substantial increase in the stability of the $R_{3\times 3}$ phase. The main part of Figure 15c shows that the $R_{3\times 3}$ phase is present at temperatures up to 0.27, and the $R_{5\times 5}$ phase appears only at higher temperatures, between 0.28 and about 0.32. Again, the partially ordered LAD phase appears only on descending isotherms, and over a rather limited temperature range, between 0.16 and 0.20. The inset to Figure 15c presents the isotherms recorded at T=0.16 and 0.14. The descending isotherm at t=0.16 shows a plateau at ρ≈0.8, which marks the presence of the LAD phase. On the other hand, the descending isotherm at =0.14 shows quite different behavior, and the plateau occurs at a density of ρ=0.75. The inspection of snapshots showed that the film structure corresponds to the Kagome lattice (see the snapshot in Figure 16). This K2 phase has the energy (per particle) equal to

(5) $$u_{K2}=\left(11/12\right)u_{AA}+u_{AB}/6+\left(11/12\right)u_{BB}\;\;,$$

and is not stable in the ground state for any $u_{AB}$, when $u_{AA}=-1.0$ and $u_{BB}=0$.

It can be readily shown that the K2 phase is metastable, and does not appear in the ground state. Of course, the K2 phase is expected to become stable when $u_{BB}$ becomes attractive since such systems become similar to the model of particles with two attractive patches, which are known to order into the Kagome lattice [7].

Now, we would like to make some comments regarding the changes in the behavior of systems with different $u_{AB}$. In particular, we consider the changes in the stability of the $R_{3\times 3}$, $R_{5\times 5}$, and LAD phases, which depend on the differences between their energies. In the ground state, the energy (per site) of the $R_{3\times 3}$ phase is independent of $u_{AB}$, and equal to −0.5, while the energies of the $R_{5\times 5}$ and LAD phases gradually increase with $u_{AB}$ (cf. Table 1). The energy of the $R_{5\times 5}$ phase weakly depends on $u_{AB}$, and changes from −0.52, when $u_{AB}=2.0$, to −0.44, when $u_{AB}=3.0$. On the other hand, the energy of the LAD phase shows a considerably stronger dependence on $u_{AB}$, and changes from −0.6, when $u_{AB}=1.5$, to −0.3, when $u_{AB}=3.0$.

At finite temperatures, the entropic effects come into play, and their role increases with temperature. Nonetheless, energetic contributions can be expected to dominate at low temperatures and promote the formation of ordered phases over certain ranges of μ. For example, when $u_{AB}=1.5$, the energy of the LAD phase is low enough to ensure its stability over a rather wide range of temperatures (cf. Figure 14a). In this system, the OL phase also appears. By increasing $u_{AB}$ to 2.0, the energies of the OL, $R_{3\times 3}$, and LAD phases become the same, and equal to −0.5, while the energy of the $R_{5\times 5}$ phase is slightly lower and equal to −0.52. In this particular case, the OL and $R_{3\times 3}$ phases are not stable in the ground state. The calculations have shown that these phases do not appear at finite temperatures as well. On the other hand, the $R_{5\times 5}$ phase is stable at low temperatures (see Figure 15a).

**Figure 17:**
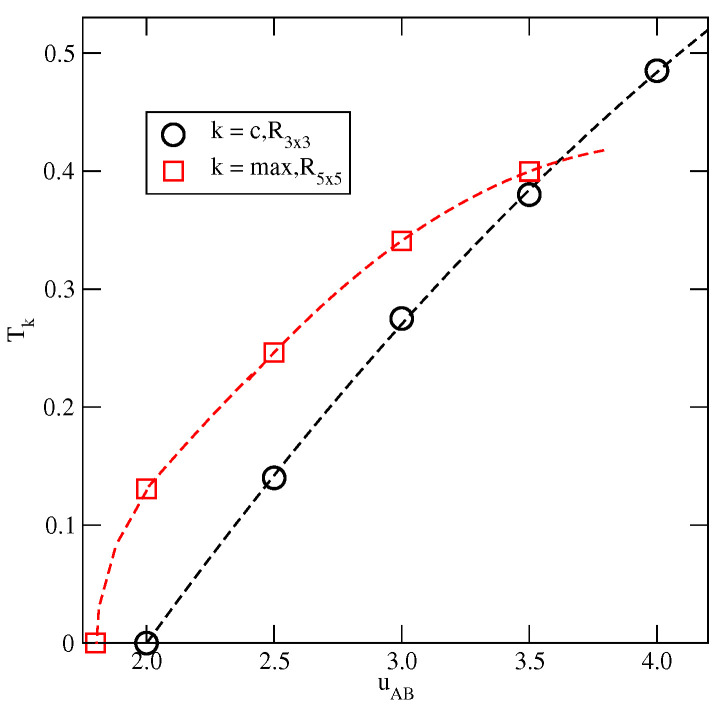
The changes in $T_{c,R_{3\times 3}}$ and $T_{max,R_{5\times 5}}$$u_{AB}$.

For $u_{AB}$ larger than 2.0, the $R_{3\times 3}$ phase develops via a discontinuous transition from the low-density cluster gas. The results suggest that the $CG-R_{3\times 3}$ transition terminates at the critical point, $T_{c,R_{3\times 3}}$, which is located at gradually increasing temperature, when $u_{AB}$ becomes higher (see Figure 17). At the temperatures below the critical point, the $R_{5\times 5}$ phase does not appear at all.

**Figure 18:**
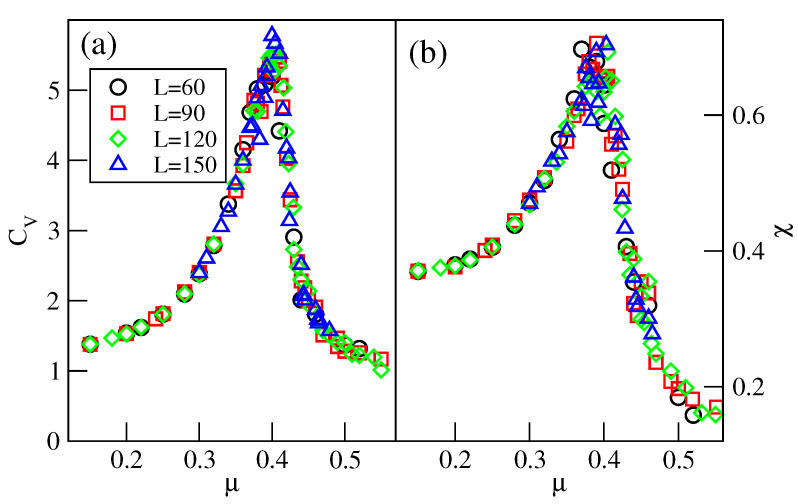
Isothermal changes in the heat capacity (**a**), and the density susceptibility (**b**), for the system with $u_{AB}=3.0$, at T=0.28, and for different sizes of the simulation cell (given in the figure).

Over a small interval of temperature, above the critical point of the $CG-R_{3\times 3}$ transition, the system is not well-ordered and exhibits small domains characteristic of both the $R_{3\times 3}$ and $R_{5\times 5}$ phases (see Figure S6). The well-defined $R_{5\times 5}$ phase shows up only at still higher temperatures. However, the recorded isotherms demonstrate that the $R_{5\times 5}$ phase develops gradually from the disordered low-density fluid. To verify this observation, we calculated the heat capacity and the density susceptibility for the system with $u_{AB}=3.0$, at the temperature of 0.28, and using the simulation cells of different sizes *L*, between 60 and 150. The results, given in Figure 18, demonstrate that these two quantities do not show finite size effects expected for continuous phase transitions [29].

The $R_{5\times 5}$ phase remains stable at temperatures up to $T_{max,R_{5\times 5}}$, and the estimated values of $T_{max,R_{5\times 5}}$ are shown in Figure 17. It should be noted that a precise localization of $T_{max,R_{5\times 5}}$ is difficult since the defected $R_{5\times 5}$ phase exists over a certain range of temperature. Therefore, the values of $T_{max,R_{5\times 5}}$ given in Figure 17 may be slightly underestimated. Nonetheless, the results demonstrate that the $R_{5\times 5}$ phase should not appear when $u_{AB}$ becomes larger than about 3.6. We performed simulations for $u_{AB}=4$, which confirmed the lack of the $R_{5\times 5}$ phase, at the temperatures above $T_{c,R_{3\times 3}}$.

## 4. The Model and Methods

As already mentioned, the model applied is the same as used in Refs. [13,14]. The Janus-like particles, composed of parts A and B, are placed on a triangular lattice and allowed to take on one of the six orientations, defined by the angle θ(k)=(k−1)(2π/6) (k=1,…,6), measured with respect to the *x*-axis of the lattice. Throughout this work, we assumed that all interactions are limited to the first nearest neighbors.

The spin vector of unit length, S=(cos(θ),sin(θ)) is assigned to each particle, and the energy of interaction between a pair of particles on adjacent sites, *i* and *j*, can be represented as $u(S_{i},S_{j},r_{ij})$, where $r_{ij}$ is the separation vector of unit length. Moreover, we assumed that $u(S_{i},S_{j},r_{ij})$ depends on the degree to which the parts A and B face each other; hence, $u(S_{i},S_{j},r_{ij})$ can be written as

(6) $$u\left(S_{i},S_{j},r_{ij}\right)=w_{AA}\left(S_{i},S_{j},r_{ij}\right)u_{AA}+w_{AB}\left(S_{i},S_{j},r_{ij}\right)u_{AB}+w_{BB}\left(S_{i},S_{j},r_{ij}\right)u_{BB}\;.$$

In the above equation $w_{AA}\left(S_{i},S_{j},r_{ij}\right)$, $w_{AB}\left(S_{i},S_{j},r_{ij}\right)$, and $w_{BB}\left(S_{i},S_{j},r_{ij}\right)$ are the weights, determined by the degrees to which the AA, AB, and BB regions overlap, for given orientations of the particles and the separation vector. There are 12 different values of the pair energy, which can be found in Table I of Ref. [18].

With the above assumptions, the Hamiltonian of the model reads

(7) $$H=\frac{1}{2}\sum_{i,j}u\left(S_{i},S_{j},r_{ij}\right)n_{i}n_{j}-N\mu \;\;,$$

where the sum runs over all pairs of nearest neighbors and $n_{i}$ is the occupation variable, equal to 1 (0). When the *i*-the site is occupied (empty), N is the number of particles, and μ is the chemical potential. For the system of linear dimension *L*,

(8) $$N=\sum_{i=1}^{L^{2}}n_{i}\;,$$

and the total density is equal to $\rho =N/L^{2}$. The densities of differently oriented particles are given by

(9) $$\rho_{k}=\frac{1}{L^{2}}\sum_{i=1}^{L^{2}}n_{i}\delta \left(\theta \left(k_{i}\right)-\theta \left(k\right)\right).$$

The AA interaction is attractive and fixed, with $u_{AA}=-1.0$, while $u_{AB}$ and $u_{BB}$ are repulsive and varied. The temperature, chemical potential, and all energy-like quantities have been expressed in the reduced units of $|u_{AA}|$.

To study the phase behavior and self-assembly, we used the Monte Carlo method in the grand canonical ensemble [30]. The simulations were performed using rhomboid cells of the size L×L, with standard periodic boundary conditions. Since the ordered structures with different unit cells and symmetry can appear, the size of the simulation box had to be properly chosen to accommodate them in periodically repeated simulation cells.

The main quantities recorded included the averages of total density, 〈ρ〉, and densities of differently oriented particles, $\langle \rho_{k}\rangle$, the potential energy per site, 〈u〉, the heat capacity

(10) $$C_{V}=\frac{1}{T^{2}L^{2}}\left[\langle H^{2}\rangle -{\langle H\rangle }^{2}\right]$$

and the density susceptibility

(11) $$\chi_{\rho }=\frac{1}{T}\left[\langle \rho^{2}\rangle -{\langle \rho \rangle }^{2}\right].$$

To equilibrate the system, $10^{6}$–$10^{7}$ Monte Carlo steps were used, and another $5\times 10^{6}$–$10^{8}$ Monte Carlo steps were performed to calculate averages. Each Monte Carlo step involved $10\cdot L^{2}$ attempts to change the state of the system. In the grand canonical ensemble, the possible changes in the system state involved either a creation of particle on a randomly chosen site, with also randomly chosen orientation, or a removal of a randomly chosen particle. The simulation at a given temperature usually started at the low value of the chemical potential, corresponding to a very low density. Then, the chemical potential was gradually increased, up to the values at which the entire lattice was nearly filled. After the recording of such “ascending” isotherm, we performed the run, starting at high chemical potential (and density), and recorded “the descending” isotherm. This procedure allowed us to estimate the locations of first-order phase transitions. In finite systems, any first-order transition is usually accompanied by a hysteresis loop due to the presence of long-living metastable states [26,30]. During the equilibration runs, the changes in the recorded quantities were monitored, and the equilibration was assumed to be complete when these quantities showed only oscillations around the average values. In some cases, equilibrium was already achieved after $10^{5}$–$5\times 10^{6}$ Monte Carlo steps, but usually, it required a larger number of Monte Carlo steps, up to $10^{7}$.

To determine the range of temperature over which a given ordered phase is stable, simulations in the canonical ensemble were used. The runs started at very low temperatures with the configurations corresponding to perfectly ordered phases. The attempted moves involved the change of orientation or a jump of a randomly chosen particle to a neighboring site.

## 5. Conclusions

We discussed the phase behavior of Janus-like particles on a triangular lattice, assuming that interactions between their parts A and B are orientation-dependent. It was assumed that only the AA interaction is attractive, while the AB and BB interactions are repulsive. In such systems, the formation of clusters with possibly the largest number of AA contacts is favored and leads to the formation of several ordered phases of different density and symmetry, as shown in Figure 2. This demonstrates that the relative strength of the repulsive AB and BB interactions is important in the self-assembly processes of Janus particles.

Three series of systems were studied. In the first series, the AB and BB interactions were assumed to be the same, with equally disfavored AB and BB contacts. The second (third) series involved systems in which $u_{AB}=0$ and $u_{BB}>0$ ($u_{BB}=0$ and $u_{AB}>0$).

The estimated phase diagrams for the first two series (cf. Figure 5, Figure 9, and Figure 10) demonstrated the formation of all ordered phases predicted by the ground state calculations. In many cases, the phase diagrams exhibited re-entrant behavior, since the ordered phases showed the largest stability when perfectly ordered. It should be stressed that the canonical ensemble simulations confirmed that the regions of stability of different ordered phases are the same as found in the grand canonical simulations. The re-entrant behavior can be attributed to the growing importance of entropic effects with temperature. However, direct transitions between different ordered phases take place at low temperatures. This gives rise to the appearance of triple points (cf. Figure 5, Figure 7, and Figure 8).

The systems with $u_{BB}=0$ and $u_{AB}>0$ were found to exhibit more complex phase behavior, and only for $u_{AB}$ up to 1.5. The results of simulations at finite temperatures were consistent with the ground state predictions. The behavior of systems with $u_{AB}>2$ was rather peculiar since it did not seem to agree with the ground state predictions. In particular, only one of the $R_{3\times 3}$ and $R_{5\times 5}$ phases appeared at any given temperature. It was suggested that it resulted from the changes in the structure of the disordered low-density gas-like phase with temperature. In addition, the adsorption isotherms never showed the expected LAD phase. The defected LAD phase appeared only on desorption isotherms.

Our results also demonstrated that disordered phases may be stable even at quite low temperatures, pointing to the role of entropic effects. Although the ordered phases were degenerated, their stability was primarily determined by energetic effects. On the other hand, the disordered states were characterized by large configurational entropy, and the entropic effects could far outweigh the energy effects. Here, we should mention the work by Smallenburg and Sciortino [31]. These authors showed that the limited valence of patchy particles may lead to situations in which the liquid phase may be stable even when the temperature goes to zero. In our model, the valence was also low, due to repulsive AB and BB interactions.

Another interesting result is the formation of structure with kagome lattie (K). Such structures have been observed by experimental [7], theoretical [32], and simulation [33,34] studies on the self-assembly of triblock patchy particles, in which two attractive patches are located at the opposite poles of particles. The possibility of the formation of an ordered phase with the same structure as shown in Figure 2j, in the system of Janus particles built from two parts, was shown in one of our earlier works [14]. In that paper, we used a similar lattice model with $u_{AB}=u_{BB}=0$ and assumed the same strength of AA interaction for all mutual orientations of neighboring molecules in which their A parts overlap. Thus, the potential used was similar to that proposed by Kern and Frenkel [15]. This ordered *K* phase was found to be stable over a considerably larger range of temperatures than observed in the present paper. In addition, the increase in chemical potential led to a gradual increase in density and the formation of the high-density mosaic structure (cf. Figure 14 in Ref. [14]), rather than to the LAM phase.

Although the considered lattice model has no analog in any known real systems, it nevertheless shows that orientation-dependent interactions between the particles can lead to the formation of a variety of ordered phases. Of course, the model used can describe the processes leading to the formation of monolayer films on crystalline surfaces when the potential barriers between adjacent adsorption sites are sufficiently high [35]. This question has not been considered.

Althought we are not aware of any real spherical Janus particles with repulsive AB and BB interactions, it is nevertheless possible that appropriate modification of the Janus particle surface may allow the formation of such systems, and the structures similar to those described in the work. The commonly used models of patchy particles [15,36,37] assume attractive AA interactions and hard-sphere potential to represent the AB and BB interactions. Repulsive orientation-dependent interactions have been considered, but only in the case of particles with shape-anisotropy (for a review, see [38]).

## Figures and Tables

**Table 1:** The energies (per particle) and the densities of ordered structures emerging from the model.

Structure (α)	$u_{\alpha }$	$\rho_{\alpha }$
OT	$\left(3/4\right)u_{AA}$	4/9
OR	$u_{AA}+\left(1/8\right)u_{AB}+\left(1/8\right)u_{BB}$	1/2
*S*	$\left(15/14\right)u_{AA}+\left(3/14\right)u_{BB}$	7/12
OL	$\left(5/4\right)u_{AA}+\left(1/4\right)u_{AB}+\left(1/2\right)u_{BB}$	2/3
$OL_{1}$	$\left(5/4\right)u_{AA}+\left(1/4\right)u_{BB}$	2/3
$R_{3\times 3}$	$\left(3/4\right)u_{AA}$	2/3
$R_{5\times 5}$	$\left(17/18\right)u_{AA}+\left(1/9\right)u_{AB}$	18/25
$OL_{3}$	$\left(2/3\right)u_{AA}+\left(1/6\right)u_{BB}$	3/4
LAD	$\left(9/8\right)u_{AA}+\left(1/4\right)u_{AB}$	4/5
*K*	$\left(5/4\right)u_{AA}+\left(1/2\right)u_{AB}+\left(3/4\right)u_{BB}$	6/7
LAM	$\left(5/4\right)u_{AA}+\left(1/2\right)u_{AB}+\left(5/4\right)u_{BB}$	1.0
$O_{4}$	$\left(3/8\right)u_{AA}+\left(9/4\right)u_{AB}+\left(3/8\right)u_{BB}$	1.0

## Data Availability

Data is contained within the article or Supplementary Material.

## Supplementary Materials

The following supporting information can be downloaded at: https://www.mdpi.com/article/10.3390/molecules29215215/s1.
