# Supplementary material for: The Ordered Structures Formed by Janus-like Particles on a Triangular Lattice
Source: Molecules. 2024 Nov 4;29(21):5215. doi: 10.3390/molecules29215215 (PMC11547329; doi:10.3390/molecules29215215)
Supplement: Supplementary file 1 [file molecules-29-05215-s001.zip › molecules-3219509-supplementary.pdf]

The oredered structures formed by Janus-like particles on a triangular lattice

A.Patrykiejew

October 24, 2024

Figure S1

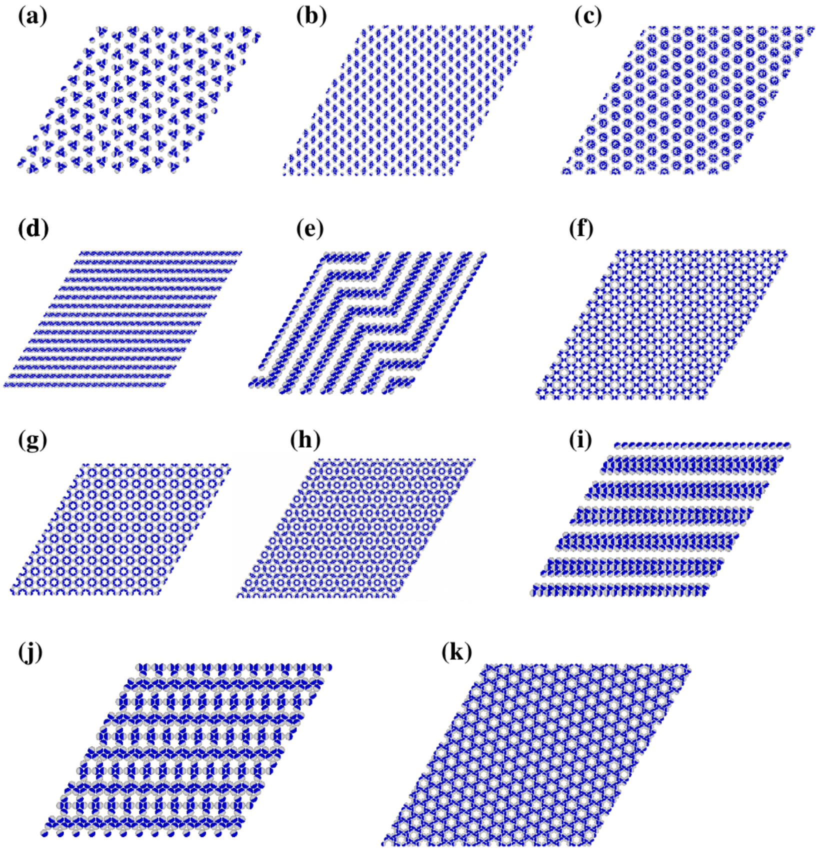

Figure S1: The ordered structures: OT (a), OR (b) S (c), OL (d), OL_1 (e), $R_{3\times3}$ (f) and (g), $R_{5\times5}$ (h), OL_3 (i), LAD (j), and K (k). A and B halves of particles are shown in dark and light blue, respectively. Note that difference in orientations of particles in parts (f) and (g).

Figure S2

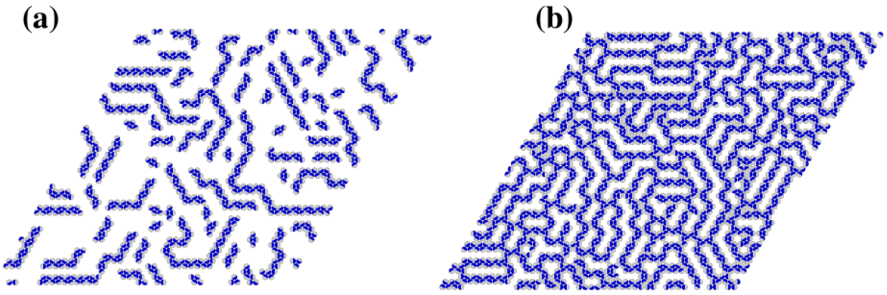

Figure S2: The examples of configurations showing the LG (a) and the LF (b) phases. These snapshots have been recorded for the system with $u^* = 0.2$, at $T = 0.16$, and $\mu = -0.80$ (a), and $\mu - 0.38$ (b).

Figure S3

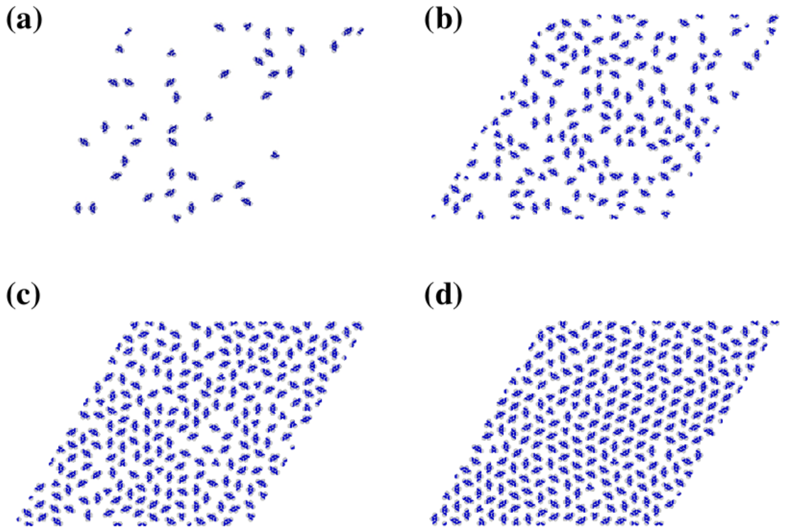

Figure S3: The examples of configurations showing the changes of cluster fluid with density. These snapshots have been recorded for the system with $u^* = 0.8$, at $T = 0.10$ and $\mu = -.78$ (a), -0.7 (b), -0.54 (c) and -0.42 (d).

Figure S4

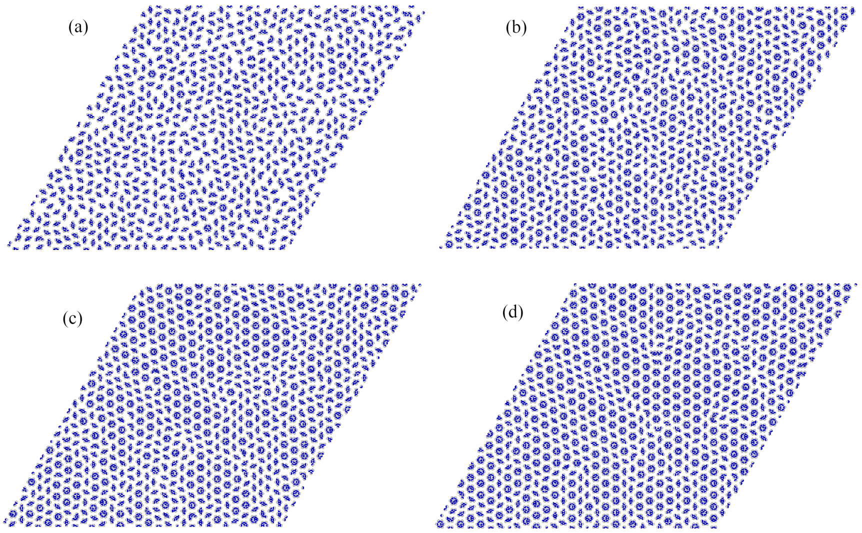

Figure S4: The configurations for the system with $u_{BB} = 1.0$, recorded at $T = 0.04$ and for the densities 0.435 (a), 0.493 (b) 0.522 (c), and 0.542 (d).

Figure S5

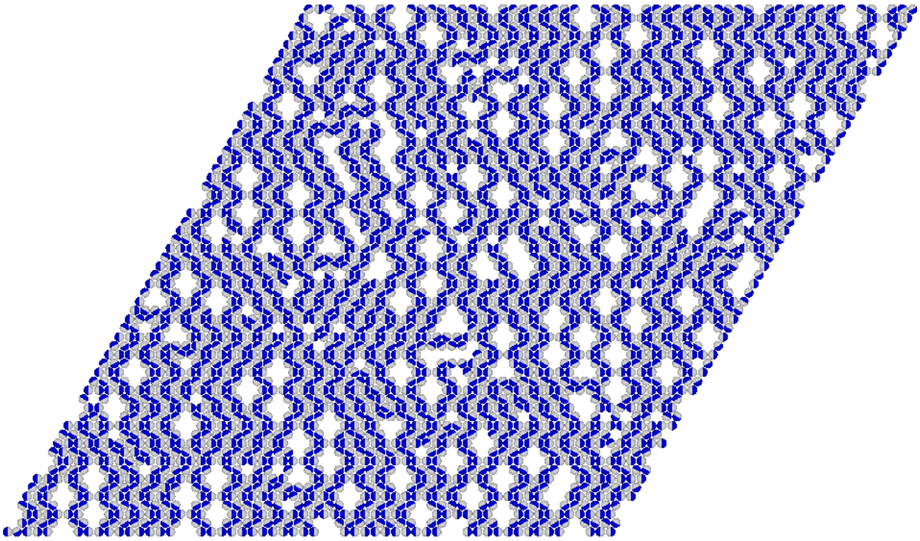

Figure S5: The snapshot recorded at $T = 0.14$ and $\mu = -0.49$ for the system with $u_{BB} = 0$ and $u_{AB} = 0.5$.

Figure S6

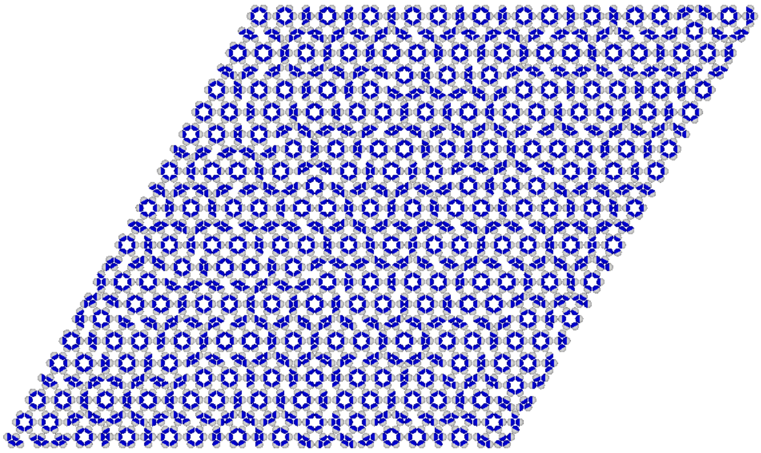

Figure S6: The snapshot recorded for the system with $u_{BB} = 0$ and $u_{AB} = 2.5$ at $T = 0.16$ and $\mu = 1.5$.
